# COVID-19 vaccine-associated myositis: a comprehensive review of the literature driven by a case report

**DOI:** 10.1007/s12026-023-09368-2

**Published:** 2023-03-16

**Authors:** Vasiliki Syrmou, Christos Liaskos, Niki Ntavari, Konstantinos Mitsimponas, Theodora Simopoulou, Ioannis Alexiou, Marianna Vlychou, Christina G. Katsiari, Dimitrios P. Bogdanos

**Affiliations:** 1 Department of Rheumatology and Clinical Immunology, Faculty of Medicine, School of Health Sciences, University General Hospital of Larissa, University of Thessaly, 41110 Larissa, Greece; 2 Department of Dermatology, Faculty of Medicine, School of Health Sciences, University General Hospital of Larissa, University of Thessaly, 41110 Larissa, Greece; 3 Department of Oral and Maxillofacial Surgery, James Cook University Hospital, South Tees NHS Trust, TS4 3BW Middlesbrough, UK; 4 Department of Radiology, Faculty of Medicine, School of Health Sciences, University General Hospital of Larissa, University of Thessaly, 41110 Larissa, Greece

**Keywords:** COVID-19 vaccine, Myositis, Dermatomyositis, Interstitial lung disease

## Abstract

Several cases of vaccine-associated manifestations have been published including cases of inflammatory myositis. Herein, we comprehensively review the literature on the occasion of case of a woman with inflammatory myositis following COVID-19 vaccination. A 67-year-old woman presented with left arm edema, rash, and weakness after the 2^nd^ dose of the BTN162b2 vaccine. Raised muscle enzymes and inflammatory markers with muscle edema on MRI and myositis findings on the electromyogram established the diagnosis. She was successfully treated with methylprednisolone pulses, intravenous immunoglobulin, methotrexate, and hydroxychloroquine. Cases of inflammatory myositis, dermatomyositis, or interstitial lung disease with myositis-specific autoantibodies or myositis-associated autoantibodies within 12 weeks from SARS-CoV-2 vaccination were included. Cases with malignancy, prior or subsequent COVID-19 infection, preexisting myositis/interstitial lung disease (ILD)/dermatomyositis (DM), or other connective tissue diseases were excluded. From our search, 49 cases were identified (mean age: 56.55 + 17.17 years), 59% were women, while 12 patients received the ChAdOx1 vaccine, 27 the BNT162b2, 8 the mRNA-1273, 1 the DB15806, and 1 the Ad26.COV2.S (overall, 70% received mRNA vaccines). Muscle involvement was the most common manifestation (79.5%), followed by skin involvement (53%) and ILD (34.6%), which were more common in the m-RNA vaccinees. Muscle biopsy, MRI findings, and autoantibody profile varied significantly, while successful immunosuppressive treatment was applied in most cases. Inflammatory myositis after COVID-19 vaccination has been well documented worldwide. Current evidence in support of a pathogenic link is challenging due to significant variation in clinical manifestations, radiological, histopathological, and immunological features.

**Supplementary Information:**

The online version contains supplementary material available at 10.1007/s12026-023-09368-2.

## Introduction

Severe acute respiratory syndrome coronavirus 2 (SARS-CoV-2) outbreak in 2020 reached rapidly the dimensions of a pandemic. From the very first months of the outbreak, huge interest around this new virus was observed and immense effort was spent in research in order to create potent and safe vaccines. By the end of the same year, vaccines had been developed by several pharmaceutical companies using not only conventional but also novel pioneer mRNA technology. Currently, the European Medicines Agency has approved 2 mRNA vaccines (Pfizer-BioNTech BNT162b2 and Moderna mRNA-1273) and 2 adenoviral vector vaccines (Oxford–AstraZeneca ChAdOx1 nCoV-19 and Janssen Ad26.COV2.S) which are in use. The safety of the vaccines has been proved to be acceptable, though occasional cases of immune-mediated adverse reactions have been described.

Overall, several cases of autoimmune phenomena, and to a lesser extent overt autoimmune disease, have been reported in patients with COVID-19 as well as in vaccinees, implying a close interplay between the virus and the host [1]. These include, but are not limited to, immune-mediated thrombotic thrombocytopenia, central nervous system demyelinating diseases, inflammatory peripheral neuropathies, myositis, autoimmune encephalomyelitis, giant cell arteritis, autoimmune hepatitis, autoimmune thyroid diseases, and autoimmune hemolytic anemia, most of which required immunomodulatory treatment. Their immunopathogenesis remains poorly understood [2, 3]. Owing to the growing use of vaccines over the globe and generalized vaccination policies and mandates, clinicians will increasingly be confronted with such infrequent adverse events.

Herein, not only we present a case of a patient that developed inflammatory myositis after the second dose of mRNA COVID-19 vaccine but more importantly we attempt to critically review the published case series of vaccine-associated myositis to assist efficacious standard-of-care approaches.

## Case presentation

**Fig. 1:**
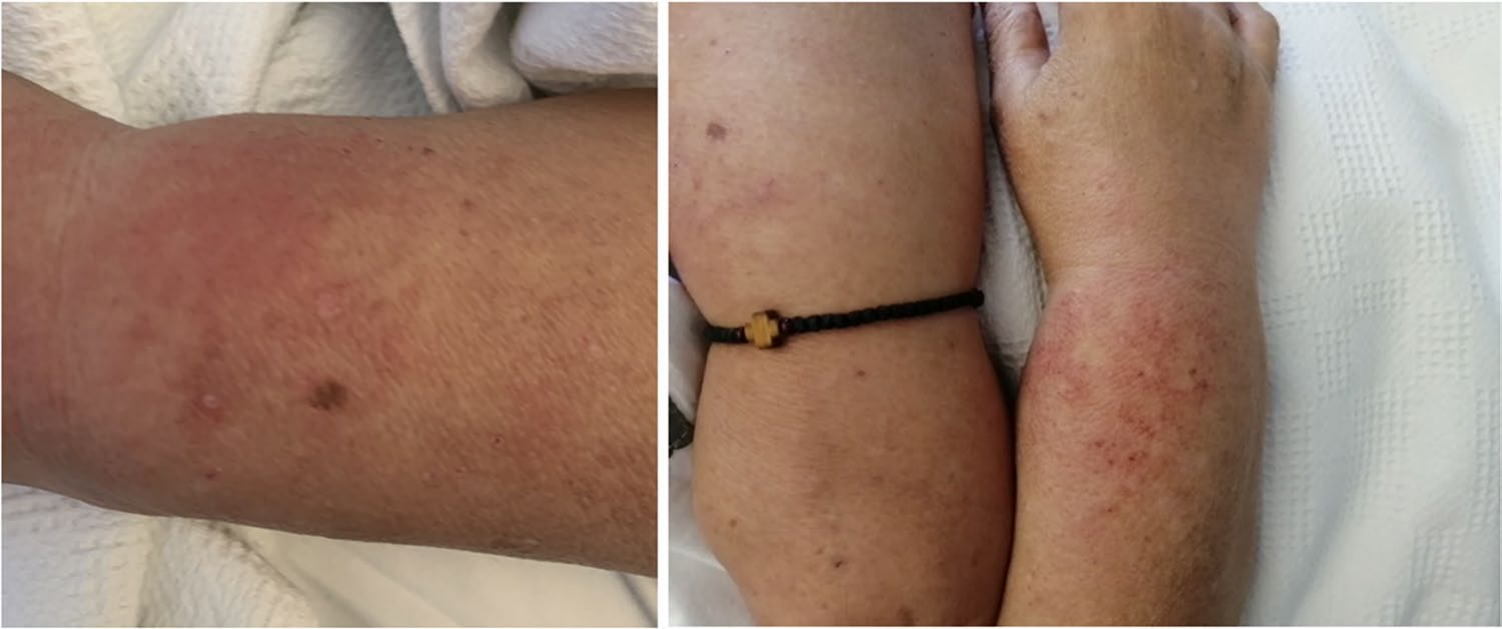
Maculopapular violaceous rash over the dorsal area of the wrists and profound unilateral edema

A 67-year-old Caucasian woman presented with 20-day history of unilateral (left) arm edema and bilateral symmetric proximal arm and leg muscle weakness. The patient reported that 2 days after receiving the 2nd dose of the mRNA COVID-19 vaccine (BTN162b2, BioNTech, Pfizer) at her left deltoid muscle, she noticed left arm edema affecting the whole limb, while she also experienced progressively worsening muscle pain and proximal weakness involving both upper and lower limbs. At the same time, she became aware of a pruritic maculopapular rash over the front area of the neck, chest, and dorsal area of the wrists (Fig. 1). The patient denied any history of either arm injury or strenuous muscle activity, while neither fever nor purulent discharge around the vaccine’s injection site was noticed. Her past medical history included the thalassemia trait, hypertension, cholecystectomy, and breast cancer treated with right mastectomy with or without lymph node dissection, chemotherapy and radiotherapy seventeen years ago. No new medications were recently initiated.

The patient was initially investigated in the context of primary care, and the basic laboratory tests revealed mild increase in creatine phosphokinase (CPK) (830 U/L, 38–190) and aspartate aminotransferase (AST) (83 IU/L, < 40) while erythrocyte sedimentation rate (ESR) was 35 mm/h and C-reactive protein (CRP) was within normal limits. Left arm deep vein thrombosis was excluded with duplex Doppler ultrasound. A mammogram had no evidence of cancer recurrence. As the symptoms did not subside, the patient had her blood tests repeated, revealing further increase in muscle enzymes with CPK up to 1507 IU/L, LDH = 344 IU/L (135–214), and AST = 110 IU/L while ESR was 44 mm/h and CRP mildly raised 0.66 mg/dL (< 0.5). She was then referred to our hospital for further investigations and management. No evidence of myoglobin in urine was found. Troponin levels were not raised. The patient never tested positive for SARS-CoV-2 infection by PCR testing.

On admission, the patient had profound edema of the left arm and severe proximal arm and leg muscle weakness. Specifically, muscle strength of the deltoids was measured at 3/5; 3/5 was also the measured muscle strength of the biceps brachii bilaterally while the strength of iliopsoas and in quadriceps femoris was assessed at 4/5 bilaterally. No distal muscle weakness was found; neck flexor muscles showed normal strength while there was no erythema or evidence of infection or crepitus around the injection site.

**Fig. 2:**
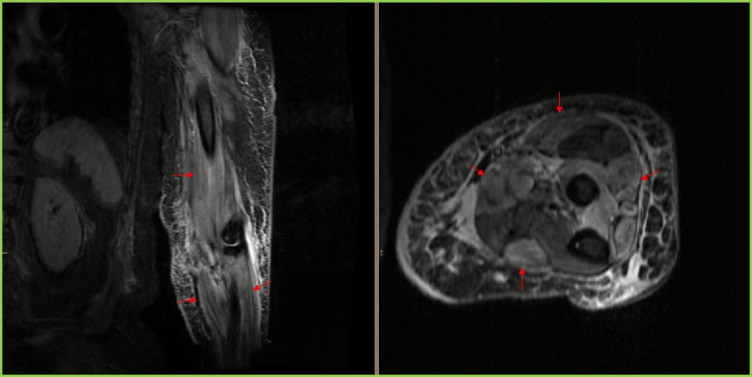
**A**, **B** Coronal STIR image of the left arm and axial T2-weighted image with fat saturation of the left forearm. Coronal STIR image of the left arm and axial T2-weighted image. There is diffuse, circumferential subcutaneous edema and increased signal of muscles, with geographic pattern of involvement of multiple compartments (arrows). Imaging findings consistent with myositis post vaccination on the left arm against SARS-CoV-2 virus

**Fig. 3:**
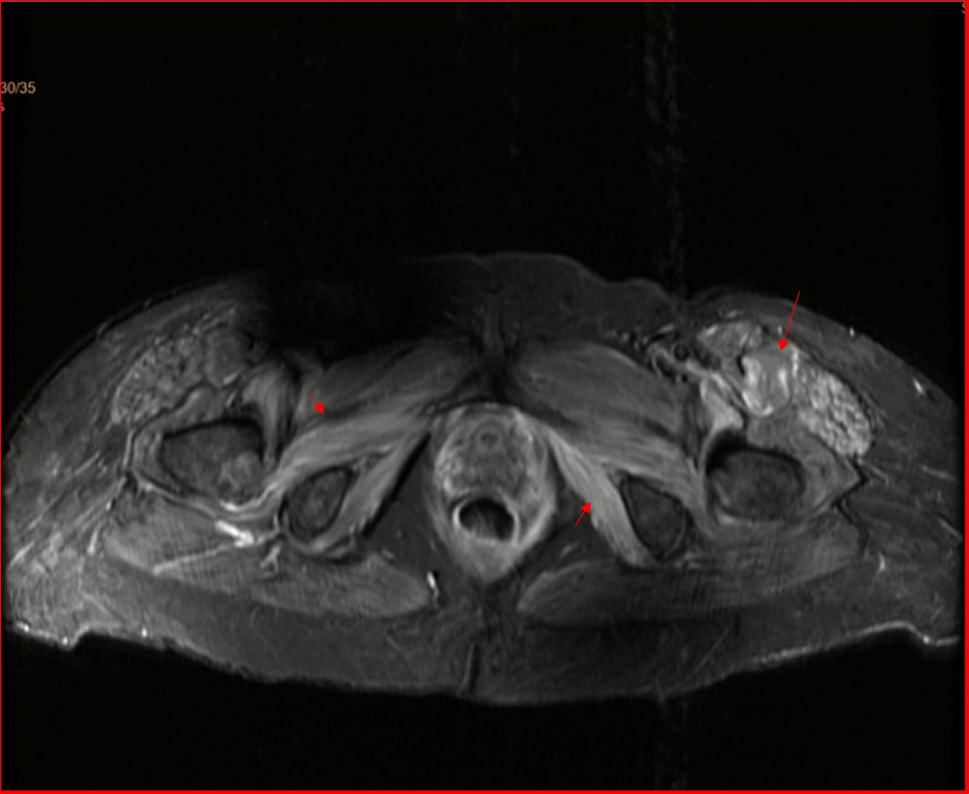
In the same patient, transverse T2-weighted image with fat saturation of the pelvis. There is increased signal of selected muscle compartments, more prominent on the left, indicative of axial skeletal myositis

Pruritic maculopapular violaceous rash over the dorsal area of the wrists was noticed (Fig. 1); no Gottron papules, heliotropic rash, or shawl sign was identified. No breast lumps or enlarged lymph nodes in axillae were palpated. The patient remained afebrile, without further increase in the inflammatory markers. Magnetic resonance imaging (MRI) of the left arm was performed ruling out abscess or other localized pathology and revealing diffusely pathologic signal distributed mainly in the muscle groups of the frontal compartment of the arm. The pathologic signal was selectively affecting some of the muscle compartments of not only the left arm but also of the left forearm, accompanied by generalized subcutaneous edema of the same areas (Fig. 2A, B). MRI of the pelvis was then performed, also revealing evidence of inflammatory myositis (Fig. 3). Electromyography revealed spontaneous activity and polyphasic potentials of short duration and low amplitude compatible with inflammatory myopathy. Muscle biopsy was not performed as the patient refused any surgical interventions. Computed tomography (CT) scan of the thorax, abdomen, and pelvis was performed without evidence of cancer recurrence. Autoantibody testing was negative for antinuclear antibodies (ANA) by indirect immunofluorescence and myositis-specific antibodies (MSA) or myositis-associated antibodies (MAA) including Mi-2α, Mi-2β, TIF1γ, MDA5, NXP2, SAE1, Ku, PM-Scl100, PM-Scl75, Jo-1, SRP, PL-7, PL-12, EJ, OJ, and Ro-52 by a line immunoassay (Euroimmun, Lübeck, Germany) and ANA-related antigens also by a line immunoassay (nucleosomes, dsDNA, histones, SS-A, Ro-52, SS-B, nRNP/Sm, Sm, Mi-2 alpha, Mi-2 beta, Ku, CENP A, CENP B, Sp100, PML, Scl-70, PM-Scl100, PM-Scl75, RP11, RP155, gp210, PCNA, and DFS70 separately) (Euroimmun).

There is diffuse, circumferential subcutaneous edema and increased signal of muscles, with geographic pattern of involvement of multiple compartments (arrows). Imaging findings are consistent with myositis post vaccination on the left arm against SARS-CoV-2 virus.

Given the clinically evident muscle weakness, elevated muscle enzymes, and characteristic EMG and MRI findings, the diagnosis of inflammatory myositis was made. Consequently, the patient received intravenous daily pulses of 1 g of methylprednisolone (3 in total) followed by oral methylprednisolone at a dose of 32 mg per day along with methotrexate (15 mg/week) and hydroxychloroquine 200 mg daily. She responded promptly with complete resolution of pain, rash, and arm edema, while there was gradual improvement in muscle power and muscle enzymes and remains without problems 9 months following this episode.

## Methods

To identify all the published cases of myositis related to COVID-19, we performed a meticulous and exhaustive literature search based on terms such as “myositis” OR “myopathy” OR “dermatomyositis (DM)” OR “interstitial lung disease (ILD)” AND “COVID-19 vaccine” OR “SARS-COV-2 vaccine” in PubMed including articles published until the end of August 2022. After screening the articles for relevance and identifying the pertinent articles, we reviewed the described cases against predetermined inclusion and exclusion criteria for our study. These criteria were as follows.

- Inclusion criteria:
  o evidence of myositis confirmed either with MRI or EMG or muscle biopsy or
  p DM confirmed with skin biopsy or
  q new-onset ILD with positive myositis-specific (MSA) or myositis-associated antibodies (MAA) with or without myopathy after COVID-19 vaccination.
- Exclusion criteria:
  o evidence of malignancy
  o cases with prior or subsequent COVID-19 infection
  o preexisting myositis/ILD/skin changes before vaccination
  o time from vaccination to symptoms exceeding 12 weeks
  o connective tissue diseases associated with myositis (SLE, systemic sclerosis)

## Results

**Fig. 4:**
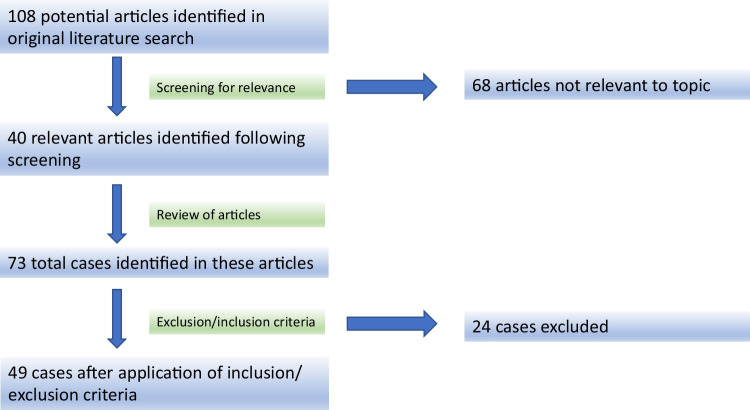
PRISMA flowchart of the literature review

One hundred and eight articles were identified that were screened for relevance. Forty were relevant to our study while 68 were irrelevant and excluded. After applying our inclusion and exclusion criteria, a total of 49 cases of confirmed myositis or amyopathic dermatomyositis or ILD post SARS-CoV-2 vaccination were finally included in our analysis (Fig. 4). The major features of each case are summarized in Tables 1, 2, and 3.

**Table 1:** Cases vaccinated with ChAdOx1 vaccine: clinical manifestations and antibody profile

Author	Skin involvement	Myositis	ILD	Antibody profile
Maramattom et al. 2021 [8]	No	Yes	No	ANA ( −), myositis profile ( −)
Maramattom et al. 2021 [8]	No	Yes	No	ANA ( −), myositis profile ( −)
Maramattom et al. 2021 [8]	No	Yes	No	ANA ( −), myositis profile ( −)
Capassoni et al. 2021	Yes	Yes	No	ANA ( +) (1:160) anti-Pm/scl-75 ( +)
Gonzalez et al. 2022	Yes	No	Yes	ANA ( +) anti-MDA-5 ( +) anti-Ro52 ( +)
Gupta et al. 2021	Yes	Yes	Yes	ANA ( −), anti-Ro52 ( +) anti-Jo1 ( +)
De Marco et al. 2022 [13]	Yes	No	Yes	ANA ( +) anti-SL 75 ( +), anti-Ro52 ( +)
De Marco et al. 2022 [13]	No	Yes	No	ANA ( +) myositis profile ( −)
De Marco et al. 2022 [13]	No	Yes	No	ANA ( −) anti-Pl12 ( +) and anti-Scl100 ( +)
De Marco et al. 2022 [13]	No	Yes	No	ANA ( +) anti-Jo1 ( +)
De Marco et al. 2022 [13]	No	Yes	No	ANA ( −), myositis profile ( −)
De Marco et al. 2022 [13]	No	Yes	No	ANA ( −), anti-SRP ( +)

**Table 2:** Cases vaccinated with mRNA-1273 vaccine: clinical manifestations and antibody profile

Author	Skin involvement	Myositis	ILD	Antibody profile
Venkateswaran et al. 2022	Yes	Yes	No	ANA ( +) 1:160 myositis profile ( −)
Carrasco et al. 2021	Yes	No	Yes	ANA ( +) 1/320 anti-MDA5 ( +)
Gonzalez et al. 2022	Yes	No	Yes	Anti-Ro-52 ( +) anti-MDA5 ( +) (168 < 15)
Faissner et al. 2021	No	Yes	No	ANA ( −) myositis profile ( −)
Kondo et al. 2022 [22]	Yes	Yes	No	ANA ( −) myositis profile ( −)
Kondo et al. 2022 [22]	Yes	Yes	No	ANA ( −) myositis profile ( −)
Kondo et al. 2022 [22]	Yes	Yes	No	ANA ( −) myositis profile ( −)
Kitajima et al. 2022	Yes	No	Yes	ANA ( −) anti-MDA5 ( +)

**Table 3:** Cases vaccinated with BNT162b2 vaccine: clinical manifestations and antibody profile

Author	Skin involvement	Myositis	ILD	Antibody profile
Theodorou et al. 2021 [6]	No	Yes	No	Unknown
Ramalingam et al. 2021 [7]	No	Yes	No	Unknown
Kaulen et al. 2021	No	Yes	No	ANA ( −) anti-PM/Scl-75 ( +)
Kaulen et al. 2021	No	Yes	No	ANA ( −) anti-SAE1 ( +)
Gouda et al. 2022	Yes	Yes	Yes	ANA ( +) 1/80 anti-RNP = 39 (< 20)
Al-Rasbi et al. 2022	No	Yes	Yes	ANA ( −)
Kim et al. 2022 [20]	Yes	Yes	No	ANA ( +) (1:160), anti-Pm/scl-75 ( +)
Vutipongsatorn et al. 2022	Yes	Yes	No	ANA ( −) anti-Mi-2a ( +) and anti-Ro-52 ( +)
Gonzalez et al. 2022	Yes	Yes	Yes	ANA ( +) 1:640 anti-MDA5 ( +)
Gonzalez et al. 2022	Yes	Yes	Yes	ΑΝΑ ( −) anti-MDA5 ( +) anti-TIF1γ ( +)
Gonzalez et al. 2022	Yes	No	Yes	ANA ( −) anti-Ro-52 ( +) anti-MDA5 ( +)
Gonzalez et al. 2022	Yes	No	Yes	ANA ( +) 1/640 anti-MDA5 ( +) anti-Ro-52 ( +)
Magen et al. 2022 [23]	No	Yes	No	ANA ( +) myositis profile ( −)
De Marco et al. 2022 [13]	No	Yes	Yes	Anti-Jo1 ( +) Ro52 ( +)
De Marco et al. 2022 [13]	No	Yes	No	ANA ( −), myositis profile ( −) anti-HMGCR +
De Marco et al. 2022 [13]	No	Yes	Yes	ANA ( −), myositis profile ( −) anti-HMGCR +
De Marco et al. 2022 [13]	No	Yes	No	ANA ( −), myositis profile ( −) anti-HMGCR +
De Marco et al. 2022 [13]	No	Yes	No	ANA + (Sm/RNP/anti-chromatin +)
Dodig et al. 2022 [24]	No	Yes	No	ANA ( +) > 1/640 anti-SRP ( +)
Wu et al. 2022 [25]	Yes	Yes	No	ANA ( −), anti-TIF-1γ ( +)
Camargo-Coronel et al. 2022 [19]	Yes	Yes	No	ANA ( −) anti-Mi2a ( +) anti-Mi2b ( +)
Kreuter et al. 2022	Yes	No	No	ANA ( +) 1/320 anti-Ro52 ( +) anti-TIF1γ ( +) anti-SRP ( +)
Holzer et al. 2022 [21]	Yes	Yes	Yes	ANA ( −) anti-MDA5 ( +) anti-Ro52
Holzer et al. 2022 [21]	Yes	No	No	ANA ( +) 1/5120 anti-MDA5 ( +)
Holzer et al. 2022 [21]	Yes	No	No	NA ( +) 1/1280 anti-MDA5 ( +) anti-NXP2 ( +)
Kitajima et al. 2022	Yes	Yes	Yes	ANA ( −) anti-MDA5 +
Kitajima et al. 2022	Yes	No	Yes	ANA ( −) anti-MDA5 +

Scrutinizing the excluded cases, there was a plethora of rhabdomyolysis cases in literature. However, none of those was included in the present study as there was no confirmation of the diagnosis of myositis with either MRI, EMG, or biopsy. Six cases of myositis in patients with concurrent malignancy, 7 cases in patients with ILD without MSA or MAA, 3 cases that developed symptoms beyond 12 weeks after last vaccination, 1 case in a patient with SLE diagnosis, and 1 case with myositis ossificans were also excluded.

Among the remaining 49 cases, a slight female preponderance was observed (female to male ratio: 59 vs. 41%) and a mean age of 56.55 + 17.17 years. Twelve patients received the ChAdOx1 vaccine, 27 the BNT162b2 vaccine, 8 the mRNA-1273, 1 patient received DB15806 (CoronaVac), and 1 the Ad26.COV2.S (Janssen). 70% of the cases were documented after the mRNA vaccines (BNT162b2 and mRNA-1273). For the ChAdOx1 vaccine, 5 cases were documented after the 1^st^ dose (average time from vaccination to symptoms initiation 12 days) and 6 cases after the 2^nd^ dose (i.e., 3 months later—time between doses—plus average time from vaccination to symptoms 22.5 days). In one case, no information was provided regarding the dose. For the BNT162b2 vaccine, there were 10 cases after the 1^st^ dose (average time 14.8 days), 12 cases after the 2^nd^ dose (i.e., 3 weeks later—time between doses—plus average time 14.16 days), and 3 cases after the 3^rd^ (average time 32.6 days, undefined time frame from previous doses) while for 2 patients no information was provided regarding the vaccine dose. In the mRNA-1273 group, 2 cases were documented after the 1^st^ dose (average time 3 days), 4 cases after the 2^nd^ dose (i.e., 3 weeks later—time between doses—plus average time 4.5 days after the dose) while there were 2 cases where no further information was provided. Regarding the Ad26.COV2.S vaccine, the patient developed symptomatology 10 days after the 1st dose of vaccination [4]. As far as the DB15806 vaccine is concerned, Tan et al. (2022) [5] described one case of immune-mediated necrotizing myositis in a 54-year-old man seven days post 2nd dose of CoronaVac, who presented with calf pain, proximal muscle weakness dysarthria, and dysphagia. He had increased muscle enzymes (CPK = 27.000), anti-SRP autoantibodies, and features of myositis on EMG without evidence of malignancy on CT scan. Biopsy of the deltoid muscle revealed scattered necrotic and regenerating muscle fibers without marked inflammation. The patient was treated with oral prednisolone and IVIG.

Regarding the observed clinical manifestations, muscle inflammation (defined as muscle edema on MRI or compatible EMG or muscle biopsy with inflammation or ILD with MAA/MSA with raised muscle enzymes) was the most common finding (79.5%), affecting 77% (27 out of 35) of the mRNA vaccine cases (22 out of 27 in the BNT162b2 and 5 out of 8 in the mRNA-1273 group) and 84.6% from the adenovector vaccines (11 out of 13, 10/12 in the ChAdOx1 and the Ad26.COV2. S case).

Skin involvement (53%) was observed more commonly in the mRNA vaccines (62.8%—22 out of 35 vs. 30.7%—4 out of 13) and especially in the mRNA-1273 group (87.5%, 7 out of 8 cases).

Finally, evidence of ILD (34.6%) was identified in 40% of cases in the mRNA group (40%—14 out of 35, 11 cases in the BNT162b2 and 3 cases in the mRNA-1273 group, respectively), while in the adenovector vaccine there were 3 cases (21.4%) all in the ChAdOx1 group (23%—3 out of 13 adenovector vaccine cases).

Muscle biopsy has only been performed in a small number of patients. Hence, conclusions cannot be drawn out safely. However, there were cases with either evidence of inflammatory myositis or necrosis. Muscle biopsy has been not performed in all cases (18), and the description of the findings varied among cases, a finding rather confusing. Among those cases, 17 patients had evidence of inflammation compatible with myositis and 7 patients (4 from the BNT162b2 group, 2 from the ChAdOx1 group, and 1 with the DB15806 vaccine) had evidence of necrosis on muscle biopsy, while 3 cases had anti-SRP autoantibodies, 1 anti-HMGCR, 1 anti-TIFγ, and 1 ANAs (speckled pattern). Three of the 4 cases with anti-SRP autoantibodies had evidence of necrosis on muscle biopsy (in the 4^th^ case muscle biopsy was not done). Anti-PM/Scl-75 (2 cases), anti-Mi2 (1 case), anti-(Sm/RNP/anti-chromatin +) (1 case), anti-Pl-12 + and anti-Scl100 + (1 case), and anti-HMGCR + (1 case) had more dominant myopathic features with versus skin manifestations or ILD. Finally, anti-MDA5 ( +) cases (11 out of 13) had ILD with typical skin changes (12 out of 13) and minimal or no muscle involvement. MRI findings also varied, from scattered patchy pattern and local inflammation to diffuse muscle edema.

In terms of the autoantibody profile status, analysis was performed for ANA and relevant myositis profile. For two cases, no information was provided [6, 7]. ANAs were found positive in 17 cases (36%, 10 cases in the BNT162b2 group, 5 cases in the ChAdOx1 group, and 2 cases in the mRNA-1273 group). Thirteen cases tested positive for anti-MDA5 (27.6%, 11 cases in the BNT162b2, 3 in the mRNA-1273, and 1 in the ChAdOx1 group), 8 of them presenting with ILD. Anti-Ro52 was found positive in 10 cases (21%), anti-SRP in 4 cases (3 with evidence of necrosis on muscle biopsy), anti-TIF1γ in 3 cases (all in the BNT162b2 group with extensive skin manifestations), and 3 cases with anti-HMGCR antibodies (all in the BNT162b2 group and all on statins). The remaining cases with a positive myositis profile included individuals with anti-Jo1 (3), anti-SAE1 (1), anti-NXP2 (1), anti-PM/Scl75 (3), anti-Mi2a (2), antiMi2b (1), anti-RNP (1), and anti-Scl100 (1) antibodies.

In terms of management, these cases were treated taking into account the clinical severity, as per standard of care in IIM treatment. High-dose steroids were applied avidly except 4 cases (2 with amyopathic DM, 1 anti-HMGCR + with mild myopathy, and 1 with local inflammation limited to the deltoid muscle). Most cases required i.v. glucocorticoids in the initial management. Intravenous immunoglobulin was used in 13 cases, cyclophosphamide in 7 cases, rituximab in 5 cases, mycophenolate mofetil in 6 cases, azathioprine in 5 cases, tacrolimus in 6 cases, methotrexate in 7 cases, hydroxychloroquine in 4 cases, tofacitinib in 3 cases, and colchicine in one patient. Three cases underwent plasma exchange, and one patient was started treatment with nintedanib. Regarding survival rates, four cases did not survive the episode (3 anti-MDA5 + with ILD).

## Discussion

Herein, we review the existing literature regarding inflammatory myositis following SARS-CoV-2 vaccination on the occasion of our case of idiopathic inflammatory myositis post vaccination with an mRNA vaccine. In that case, exclusion of malignancy was the priority, given the history of breast cancer. Other causes such as DVT, cellulitis, and septic myositis, which could lead to the predominant edema of the left arm, had to be excluded. However, there was no clinical evidence of infection and inflammatory markers remained low.

MRI scan of other areas of the body, away from the injection site, was performed, indicating inflammatory myositis. This finding was in agreement with previous cases [8]. The symptoms can potentially resolve spontaneously; the MRI signs, however, can persist for 2 months after vaccination [6]. In this case, it was the significant muscle weakness, along with the myopathic pattern affecting all four limbs, which guided the decision towards the administration of immunomodulatory treatment, in accord to other published cases.

Myositis-associated vaccination is not a new phenomenon, as it has already been described in association with other types of vaccine. From a literature search that we performed, cases of dermatomyositis have been described post BCG [9] and HBV vaccination [10] while there are also cases of polymyositis, post HBV vaccine [11].

The conducted literature review of the reported cases with post COVID vaccination myositis identified a female preponderance (female/male ratio = 3/2), which is in agreement with the known higher prevalence of inflammatory myositis in female patients (female/male = 2–3/1 [12]). Compared to the other known post-vaccination IIM types (after HBV and after BCG vaccines), post COVID vaccination IIM appears to affect older people (mean age = 56.55 + / − 17.17 years). However, no safe conclusion can be reached here, as typically HBV and BCG are administered at a younger age.

The majority (70%) of the documented cases was associated with the mRNA vaccines (BNT162b2 and mRNA-1273). This observation could, nevertheless, be explained by the fact that these vaccines were predominantly used in several countries, and thus, more patients were exposed to them and more adverse events are anticipated. In our review, there were also 3 patients with anti-HMGCR + myositis that were receiving statins [13].

From our search, 6 cases of myositis post COVID-19 vaccination were described in patients with evidence of malignancy (Suppl. Table 1). As possible paraneoplastic phenomena, these cases were excluded from our analysis without reasonable reason other than the potential to induce confusion. However, we cannot rule out that vaccination may have expedited the manifestation of inflammatory myositis (5 out of 6 cases with mRNA vaccines).

The time frame in our analysis was arbitrarily set at 12 weeks, in order to recognize the temporal association of vaccine and myositis and minimize the risk for other possible contributing factors like other drugs or infections. This 12-week period by no means is an absolute criterion for the diagnosis of anti-COVID-19 vaccination-associated myositis and cannot be used in clinical practice. Meticulous assessment and precise configuration of the time elapsing from the initiation of the vaccination, the exact dosage in cases of multiple doses, vaccine scheduling, and the induction of symptoms and features of myositis may resolve this troubling but clinically relevant issue. In this context, it is also difficult to draw conclusions regarding the time interval between the injection and the development of myositis, as some patients developed symptoms only a few days after the first dose, while others became symptomatic weeks after the third dose. Considering the fact that different vaccine types have different dose schedules, further analysis becomes more complicated. Based on reviews of other autoimmune phenomena such as platelet factor 4 (PF4) antibody-mediated thrombotic thrombocytopenia (VITT) [14, 15], Guillain–Barre’ syndrome [8, 16], and Bell’s palsy [17, 18] associated with COVID-19 vaccines, the great majority of the cases were documented within 30 days post vaccination coinciding with the maximal host response.

Muscle biopsy would be particularly valuable in providing more information regarding the underlying cause of the disease. There is evidence that the pathological findings vary among cases. From our assessment, we identified several cases with vasculitic changes without MAA or MSA [8], cases with evidence of inflammatory myositis [2, 13, 19–22], cases with necrotic changes [13, 23], and cases with immune-mediated necrotizing myositis [24, 25]. At times, these differences in biopsy findings correlate with the presence of antigen-specific autoantibodies and the discrepancies in clinical presentation, alluding to the complexity of muscle inflammation pathogenesis.

The limited number of cases precludes us from deriving any safe conclusions regarding epidemiology, characteristics, and mechanism of vaccine-associated inflammatory myositis. It is noteworthy that MRI findings also vary from a rather geographic pattern to diffuse hyperintense signal commonly affecting not only arms but also thighs and trunk. MSA and MAA were identified in a proportion of patients. It is interesting that in the case described by Capassoni et al., the autoantibodies presented 4 weeks after the onset of symptoms. With all the limitations imposed by the relatively small number of patients, the repeating of myositis-related autoantibody screen in 4–8 weeks’ time after the onset of symptoms appears to be a valid alternative, but this needs to be validated further. At this point, it is important to mention that the phenotype in the antibody-positive cases was similar to the underlying cause associated with each autoantibody. Thus, in the anti-MDA5 + subgroup, ILD was the dominating manifestation, while in anti-SRP + cases necrotic muscle changes were observed and in anti-TIF1γ + cases skin involvement was dominant.

The underlying mechanism of myositis development post vaccination is not yet clear. It is important to mention that SARS-CoV-2 has been linked to inflammatory myositis onset. According to Saud et al. (2021) [26], several cases of myositis associated with COVID-19 infection have been published over the last 2 years, including rhabdomyolysis, dermatomyositis, paraspinal myositis, and myasthenia. More specifically, dermatomyositis cases presented not only with the typical rashes but also with less specific erythematous rashes over the extensor surfaces of limbs and trunk with symmetric proximal muscle weakness, involving both upper and lower limbs. Even a case of bulbar weakness has been described [27]. Related autoantibodies like ANA, anti-Mi2, anti-SAE1, and anti-MDA5 were identified [28]. All these cases were treated with immunosuppression, including glucocorticoids, intravenous immune globulin, cyclophosphamide, methotrexate, hydroxychloroquine, MMF, and tocilizumab. Viral infections, directly or indirectly through polyclonal activation, bystander activation, or antigen-specific-driven mechanisms such as molecular mimicry, have been considered likely mechanisms of viral and/or vaccine-induced myositis [29–31]

Of interest, immunological cross-reactivity and molecular mimicry, involving spike dominant epitopes and myositis-related auto-antigenic targets, have been considered a likely mechanism for myositis induced by COVID-19 and its relevant vaccines. Kanduc and Shoenfield (2020) [32] described a striking oligopeptide homology between SARS-CoV-2 spike glycoprotein and human and murine peptides, providing strong evidence towards immunogenicity of the virus and its spike in humans and mice. Interestingly, this peptide homology was not observed in other mammals that are not severely affected by this virus. This is important to consider in terms of appropriate animal model selection in the production of vaccines or monoclonal antibodies. However, no evidence of molecular mimicry and immunological cross-reactivity has been obtained so far.

The mRNA vaccines encode the prefusion spike glycoprotein of SARS-CoV-2 while the adenoviral vaccines are adenoviral vectors containing the gene coding spike (S) protein of the virus. mRNA vaccines can trigger immune reactions not only by coding specific antigenic epitopes (proteins) but also themselves as nucleic acids [33]. This mRNA is surrounded by nanoparticles or liposomes that keep it intact and help it escape cleavage by RNases. These particles transfer the mRNA in the cytosol by fusion to cellular membrane and endocytosis [34]. However, while in the cytosol, mRNA can bind to several pattern recognition receptors (PRRs), including Toll-like receptors (TLRs), retinoic acid-inducible gene 1(RIG-1), and melanoma differentiation-associated protein 5 (MDA5) stimulating proinflammatory cascades via type 1 interferon and transcription factor nuclear factor (NF)-kB [35]. As it is noted in animal models, the TLR4-HMGB1 pathway is holding a leading role in the pathogenesis of inflammatory myositis leading to increase in MHC-I and other proinflammatory cytokines’ expression including IL-6 and TNF-a [36, 37]. This is particularly important in genetically predisposed individuals with a hyperactive immune system [38]. Finally, some of the proposed mechanisms of myositis triggered by the spike protein involve T cell and B cell clonal expansion and subsequent production of inflammatory cytokines leading to bystander muscle injury.

## Conclusion

Inflammatory myositis induced by SARS-CoV-2 vaccination is a rare entity. The underlying mechanism is not yet clear, and further research is required to shed light in this complex entity. A deeper insight of the close interplay between SARS-CoV-2 spike-specific and self-related autoreactive responses is urgently needed to better dissect the underlying cause of vaccine-induced or vaccine-associated immune-mediated myositis.

## Supplementary Information

Below is the link to the electronic supplementary material.

Supplementary file1 (DOCX 115 KB)

## Data Availability

The data underlying this article will be shared on reasonable request to the corresponding author.

## Abbreviations

DM: Dermatomyositis
DVT: Deep vein thrombosis
CPK: Creatine phosphokinase
AST: Aspartate aminotransferase
ESR: Erythrocyte sedimentation rate
CRP: C-reactive protein
MRI: Magnetic resonance imaging
LDH: Lactate dehydrogenase
CT: Computed tomography
ANA: Antinuclear antibodies
PCR: Polymerase chain reaction
EMG: Electromyogram
MSA: Myositis-specific antibodies
MAA: Myositis-associated antibodies
SLE: Systemic lupus erythematosus
ILD: Interstitial lung disease
PF4: Platelet factor 4
