# Supplementary material for: COVID-19 vaccine-associated myositis: a comprehensive review of the literature driven by a case report
Source: Immunol Res. 2023 Mar 16;71(4):537–46. doi: 10.1007/s12026-023-09368-2 (PMC10018601; doi:10.1007/s12026-023-09368-2)
Supplement: Supplementary file 1 — Supplementary file1 (DOCX 115 KB) [file 12026_2023_9368_MOESM1_ESM.docx]

**Supplementary Table 1**. Cases of post COVID-19 vaccination myositis in patients with malignancy.

| **Study** | **Cases** | **Age/**  **sex** | **Vaccine type** | **malignancy** | **skin** | **myositis** | **ILD** | **Autoantibody profile** | **Vaccine type** |
| --- | --- | --- | --- | --- | --- | --- | --- | --- | --- |
| Blaise et al 2021 | 1 | 41/M | BNT162b2 | melanoma | no | Necrotizing myositis on biopsy | no | Negative | BNT162b2 |
| Vutipongsatorn et al | 1 | 72/F | BNT162b2 | Pancreatic adenocarcinoma | no | yes | no | anti-fibrillarin antibody (+) | BNT162b2 |
| Aimo et al 2022 | 1 | 45/F | ChAdOx1-S | Breast Ca | yes | no | no | Anti-TIF1γ (+) | ChAdOx1-S |
| Ooi et al 2022 | 1 | 44/M | mRNA- 1273 | nasopharyngeal carcinoma | yes | yes | no | Anti-TIF1γ (+) | mRNA- 1273 |
| Yoshida et al 2022 | First case | 81/F | BNT162b2 | sigmoid colon cancer | yes | yes |  | Anti-TIF1γ (+) | BNT162b2 |
|  | Second case | 87/F | BNT162b2 | Colonic mass  Elevated 19.9 | yes | yes | no | Anti-TIF1γ (+) | BNT162b2 |

*Except of Yoshida et al, who described 2 cases as per table, all other studies reported single cases; M, male; F, female
